# Widespread circulation of West Nile virus, but not Zika virus in southern Iran

**DOI:** 10.1371/journal.pntd.0007022

**Published:** 2018-12-17

**Authors:** Mazyar Ziyaeyan, Mohammad Amin Behzadi, Victor Hugo Leyva-Grado, Kourosh Azizi, Gholamreza Pouladfar, Hedayat Dorzaban, Atoosa Ziyaeyan, Sanaz Salek, Aghyl Jaber Hashemi, Marzieh Jamalidoust

**Affiliations:** 1 Department of Clinical Virology, Clinical Microbiology Research Center, Shiraz University of Medical Sciences, Namazi Hospital, Shiraz, Iran; 2 Department of Microbiology, Icahn School of Medicine at Mount Sinai, New York, New York, United States of America; 3 Department of Medical Entomology and Vector Control, Research Center for Health Sciences, School of Health, Shiraz University of Medical Sciences, Shiraz, Iran; 4 Department of Infectious Diseases, Clinical Microbiology Research Center, Shiraz University of Medical Sciences, Namazi Hospital, Shiraz, Iran; 5 University of Toronto, Toronto, Canada; DoD - AFHSB, UNITED STATES

## Abstract

West Nile virus (WNV) and Zika virus (ZIKV) are mosquito-borne viral infections. Over the past few decades, WNV has been associated with several outbreaks involving high numbers of neuroinvasive diseases among humans. The recent re-emergence of ZIKV has been associated with congenital malformation and also with Guillain–Barre syndrome in adults. The geographic range of arthropod-borne viruses has been rapidly increasing in recent years. The objectives of this study were to determine the presence of IgG specific antibodies and the genome of WNV and ZIKV in human samples, as well as WNV and ZIKV genomes in wild-caught mosquitoes in urban and rural areas of the Hormozgan province, in southern Iran. A total of 494 serum samples were tested for the presence of WNV and ZIKV IgG antibodies using ELISA assays. One hundred and two (20.6%) samples were reactive for WNV IgG antibodies. All serum samples were negative for ZIKV IgG antibodies. Using the multivariable logistic analysis, age (45+ vs. 1–25; OR = 3.4, 95% C.I.: 1.8–6.3), occupation (mostly outdoor vs. mostly indoor; OR = 2.4, 95% C.I.: 1.1–5.2), and skin type(type I/II vs. type III/IV and type V/VI; OR = 4.3, 95% C.I.: 1.7–10.8 and OR = 2.7, 95% C.I.: 1.3–5.5 respectively, skin types based on Fitzpatrick scale) showed significant association with WNV seroreactivity. We collected 2,015 mosquitoes in 136 pools belonging to 5 genera and 14 species. Three pools of *Culex pipiens* complex were positive for WNV RNA using real-time reverse transcription polymerase chain reaction (rtRT-PCR). ZIKV RNA was not detected in any of the pools. All WNV ELISA reactive serum samples were negative for WNV RNA. In conclusion, we provided evidence of the establishment of WNV in southern Iran and no proof of ZIKV in serum samples or in mosquito vectors. The establishment of an organized arbovirus surveillance system and active case finding strategies seems to be necessary.

## Author summary

In recent decades mosquito-borne viruses have reached and adapted to new habitats, and now they can be found in nearly all continents. Facilitated goods transportation, live stock exchange, people travelling more easily, and most importantly world climate alterations, might be some of the reasons for this mosquito habitat spreading. Emergence of WNV in North America, Europe, and most Mediterranean countries like Turkey, Greece and Israel is evidence of this spreading. Furthermore, emergence and re-emergence of some of these mosquito borne viruses in new areas may be accompanied with changes in their pre-known pathogenesis. Re-emergence of ZIKV in the South Pacific and America from 2007 to 2016 was accompanied with an increase in neurovirulent diseases and congenital malformations. In this study, we evaluated the presence of WNV and ZIKV via serological and genome detection in human samples and mosquitoes (viral genome analysis) from southern Iran. This region is on the coast with a warm and tropical climate suitable for inhabitation and expansion of the vectors harboring these two viruses. We caught a large spectrum of mosquitoes from these areas. After classification, we analyzed the mosquitoes’ pools for WNV and ZIKV genomic RNA. Our results showed that 20.6% of the studied human samples were IgG reactive to WNV while no antibodies against ZIKV were detected. We found WNV RNA genome in three mosquitoes’ pools. The genomic analysis was negative for ZIKV in both human and mosquito samples. Based on the results WNV is notably circulating in southern Iran; while no evidence of ZIKV infection in people or circulation in any of the vectors was observed.

## Introduction

West Nile virus (WNV) and Zika virus (ZIKV) are mosquito-transmitted viruses from the genus *Flavivirus*. The geosgraphic range of many arthropod-borne viruses (arboviruses) has increased in recent decades, due in part to changing global climatic conditions, as well as increasing global travel of both humans and domestic animals [1, 2].

In 2007 the first major, but mild, ZIKV outbreak was reported on Yap Island in Micronesia in the western Pacific Ocean [3]. Before 2007, cases of ZIKV infection were detected only sporadically with mild symptoms in humans and because of that, ZIKV has been neglected since its discovery in 1947. However, the ZIKV disease outbreak in French Polynesia during 2012–2014 was accompanied by a high prevalence of Guillain-Barre syndrome in adults [4]. In addition, the ongoing ZIKV epidemic in Brazil has been associated with congenital infection, an unusual number of neonates with microcephaly and other central nervous system malformations [5, 6]. In 2015, the ZIKV epidemic spread from Brazil to 60 other countries and territories. Yet, active local virus transmission and cases of imported ZIKV infections are occurring all over the world [7–9].

ZIKV originally circulates in a sylvatic transmission cycle between non-human primates and forest-dwelling mosquito vectors. Humans are only accidentally infected in this sylvatic cycle. But after ZIKV adaptation to an urban cycle involving humans and domestic mosquitoes, humans are the primary amplifying hosts during epidemics. Several mosquito species have been found to be naturally infected with ZIKV, including some belonging to the genus *Aedes* (Ae.), such as *Ae*. *aegypti*, *Ae*. *albopictus* and *Ae*. *Furcifer* [10, 11]. As most studies have shown, *Aedes* mosquitoes are considered the primary ZIKV vectors; however, transmission of ZIKV may involve mosquitoes of other genuses, since the virus has also been isolated from *Culex (Cx*.*) quinquefasciatus*, *Anopheles (An*.*) coustani* and many other mosquito species in nature [12–14]. The range of natural hosts and vectors could expand through the virus spread and evolution. Any countries where *Aedes spp*., especially *Ae*.*aegypti* and *Ae*. *Albopictus* mosquitoes are present, have a high potential for geographic expansion of ZIKV [11].

WNV is the most widespread member of the Japanese encephalitis virus complex in the world. This virus expanded its range from a small area of sub-Saharan Africa to almost all continents in the last 25 years [15]. Several outbreaks involving high number of neuroinvasive disease cases have been reported among humans over the past few decades [16–18]. The enzootic cycle of WNV is maintained among ornithophilic mosquitoes and birds, whereas mammals, including humans and horses, are accidental hosts and since mammals do not develop sufficient viremia to support the transmission, they are dead-end hosts. The primary mosquito vectors are members of the *Culex spp*.; mostly, *Cx*. *pipiens*, *Cx*. *univittatus*, *Cx*. *antennatus* and *Cx*. *vishnui* complex. Infection with WNV is asymptomatic in most cases, while in approximately 20% of cases, infection results in West Nile Fever (WNF) and in less than 1% of cases it results in acute West Nile Neuroinvasive Disease (WNND) [19, 20].

Iran is located in the southeast part of the Middle East and it has been extremely affected by climate changes, favoring introduction of new mosquito-borne diseases or increasing their transmission rate. A recent study shows low invasive density levels of *Ae*. *albopictus* into the Sistan and Baluchestan province, southeastern Iran [21]. There is no obstacle for this potential primary ZIKV vector population to grow and expand in the near future. Furthermore, ZIKV positive serology has been reported in our neighboring country, Pakistan [22]. Although the potential risk of ZIKV introduction to Iran exists, no studies have been done on ZIKV infections in vector mosquitoes or in humans.

Serological surveillance data suggest human and animal exposure to WNV in some provinces of Iran as well as in some of our neighboring countries [23–26]. Recently, WNV RNA has been detected in mosquitoes’ pools of *Ochlerotatuscaspius* and *Cx*.*pipiens* specimens collected from wetlands in the northwest and north of Iran respectively [27, 28].

The objectives of this study were to determine the presence of IgG specific antibodies and the genome of WNV and ZIKV in human samples, as well as WNV and ZIKV genomes in wild-caught mosquitoes in urban and rural areas of the Hormozgan province, in southern Iran.

## Methods

### Study area

**Fig 1:**
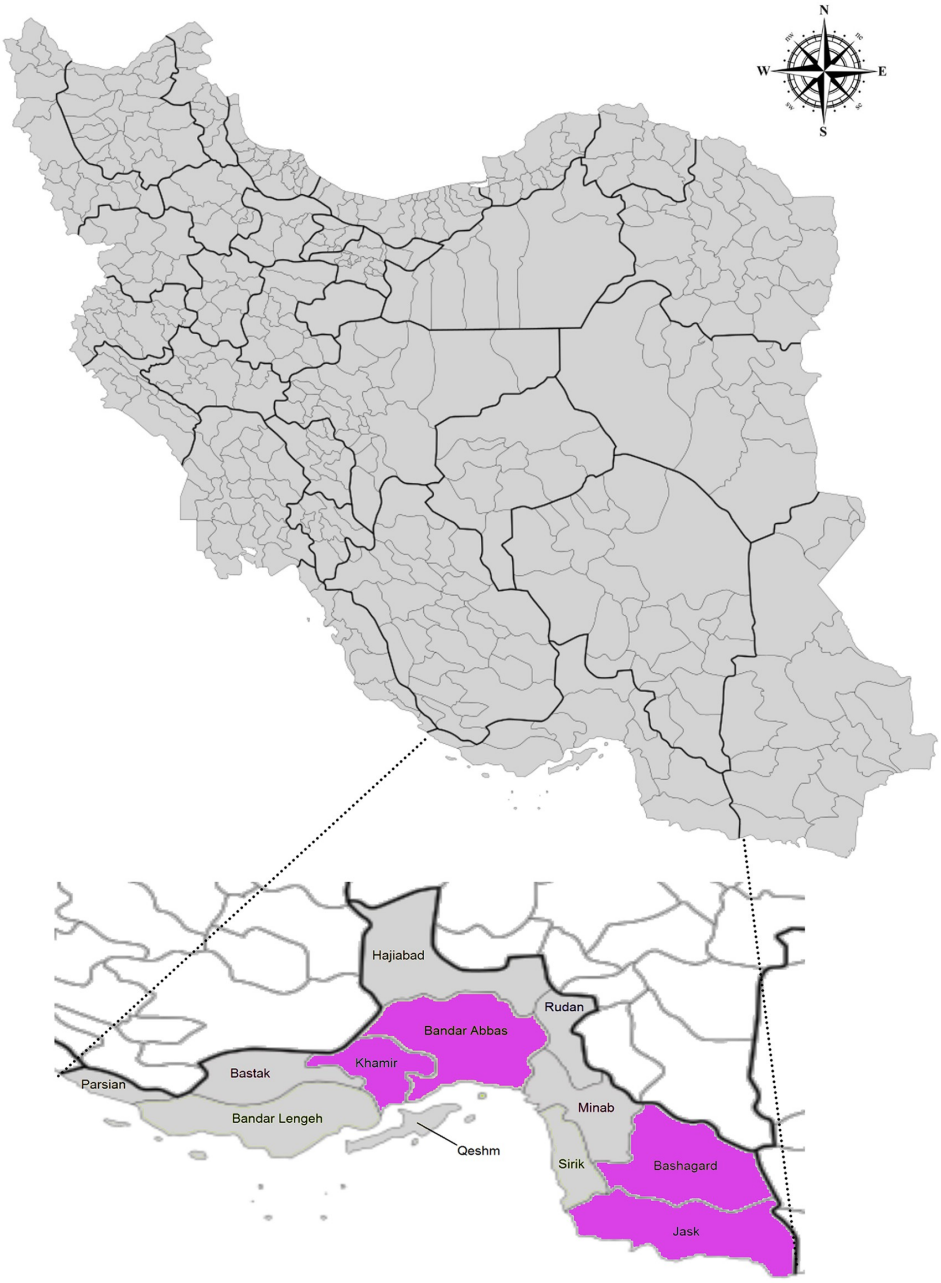
Map of Iran and Hormozgan province with location of the study counties; Bandar Abbas, Khamir, Jask and Bashagard. The sampling areas are highlighted in purple.

The study was conducted in urban and rural areas of the Hormozgan province located in southern Iran that comprises a total area of 70,697 km2 (Fig 1). Based on the 2016 general census data, there are 1.7 million people living in this province. It has hot, humid long summers and relatively arid and mild winters with limited rainfalls.

### Sample collection

From September 2016 to June 2017, a total of 494 leftover serum samples were collected after an agreement with the governmental public laboratories at four major province counties, Khamir, Bandar Abbas, Bashagard and Jask (Fig 1). Sample size was calculated by the expected proportion of positive samples, based on previous studies performed in Iran [24, 29] using 0.05 level of significance resulting in a minimum of 300 samples needed. The samples belonged to people who used the laboratory services for various purposes, for example: routine check-ups, obtaining health certificates, follow ups of their chronic diseases, etc. At the time of initial sample collection, a verbal consent was obtained from the participants. For each serum sample, basic demographic information including age, sex, place of residence, skin type or complexion and occupation was obtained from the records prepared at the time of specimen collection. The skin complexion was identified using the Fitzpatrick skin type scale that identified six different numerical classification schemes for human skin color, ranging from type I being the fairest with the lowest scores to type VI being the darkest with the highest scores [30, 31]. Based on that we categorized the skin complexion into three groups; type I/II, type III/IV, and type V/VI. This data was collected to determine if non-protected exposure of the skin to the sun and to uncovered environment has a role in susceptibility to mosquito bites. The occupations were further grouped in seven main categories: child/student, house wife, office employee, freelancer, fisherman/sailor, worker and retiree. For statistical analysis these seven groups were allocated into three major categories based on where they performed their activities: mostly indoor, usually indoor and mostly outdoor. The sera were stored at -70°C.

### Ethics statement

Approval of the study protocol was received by the Ethics Committee of the Clinical Microbiology Research Center, Shiraz University of Medical Sciences, Shiraz, Iran, which waived the need for written consent for collection of leftover sera.

### Mosquitoes

Mosquitoes were caught from several locations in four counties of Hormozgan from March 2016 to March 2017 using fixed white curtain and light traps. To minimize the sampling bias due to the increasing mosquitoes’ abundance, nets were changed every night. The insects were collected by a portable aspirator and transported to the lab where they were frozen in a −70°C freezer. The mosquitoes were placed on chilled tables and classified based on their morphological characteristics into different species and sexes. Well identified mosquitoes pooled according tocollection site, species and day of collection, into groups of one to 65 individuals, were immersed in RNase blocking solution and placed into screw-capped cryo-vials, stored and transported in liquid nitrogen gaseous phase to the Clinical Virology department at Clinical Microbiology Research Center, Shiraz University of Medical Sciences, Shiraz, Iran for virological analysis.

### ELISA tests

Commercially available ELISA kits (ZIKV Euroimmun IgG ELISA kit, catalogue number: EI 2668–9601, and WNV Euroimmun IgG ELISA kit, catalogue number: EI 2662–9601, EUROIMMUN AG, Lübeck, Germany) were used to detect IgG antibodies against ZIKV and WNV. Each commercial kit includes positive and negative control samples. The assays were carried out at the Clinical Virology department. For each sample, a ratio of the extinction value of the control or patient sample over the extinction value of the calibrator was calculated according to the manufacturer’s instructions. Specimens with a value of ≥ 1.1 were considered positive for ZIKV IgG or WNV IgG antibodies. A value of ≥ 0.8 and < 1.1 was considered as an equivocal result and a value <0.8 was determined to be negative. The negative results were further categorized into three subgroups named negative (≥ 0.51 ≤ 0.79), low negative (≥ 0.21 ≤ 0.5), and very low negative (≥ 0.05 ≤ 0.2) to reflect the different background binding levels. All samples with borderline results were tested twice and samples with positive or equivocal results were grouped as reactive.

### Indirect immunofluorescence test

All samples considered reactive for WNV IgG antibodieswere evaluated with a commercial indirect immunofluorescence test (IIFT) kit (catalogue number: FI 2662–1005, EUROIMMUN AG, Lübeck, Germany) following the manufacturer’s instructions.

### Mosquito homogenization and viral RNA extraction

Mono species mosquitoes” pool were placed in poly propylene 15-mL tubes with 2 ml of DMEM medium containing 5% fetal bovine serum. Six 4-mm diameter dense glass beads were added to each tube, and mosquito pools were homogenized by hand shaking for about 2 min and then vortexing for 30 seconds. Mosquito homogenates were centrifuged for 5 min at 2500 × g at 4°C and supernatants were collected. Viral RNA was extracted from 200 μl of each mosquitoes’ homogenate or patient sera using the High pure Viral RNA Kit (catalogue number: 11858874001, Roche Diagnostics, Mannheim, Germany) according to the manufacturer’s instructions.

### Real-time reverse transcription polymerase chain reaction (rtRT-PCR)

Commercially available rtRT-PCR primer/probe kits (Path-ZIKV-standard and Path-WNV-standard kits, Genesig, Primer design Ltd, Cambridge, United Kingdom) were used to detect and amplify ZIKV RNA and WNV RNA. All patients’ serum samples that were found positive with ELISA or IFT and all mosquitoes’ pools were subjected to the test. The tests were performed using an Applied Biosystem step one plus real-time PCR machine (Applied Biosystem, CA, USA). Amplification of ZIKV and WNV RNAs took place in a 20 μL single-tube, Superscript III Platinum one-step quantitative RT-PCR system (Catalogue number: 12574018, Invitrogen, Carlsbad, CA). Reactions contained 10.0 μL of 2X RT/PCR reaction mix, 1 μL primers/probe mix, 0.4 μL Superscript III RT/Platinum Taq mix, 0.4 μL ROX reference dye, and 5 μL of extracted sample RNA or serially diluted positive control copy number that were provided by the kits. The cycling conditions consisted of one cycle at 50°C for 30 min, one cycle at 95°C for 5 min, and 45 cycles at 95°C for 10 s and 60°C for 1 min. The test conditions for detection of WNA RNA was the same as above except for the concentration of primers/probe that was as previously described [32].

### Statistical analysis

All statistical analyses were conducted using IBM SPSS Statistics version 22 (IBM Corp, Armonk, NY). A *P*-value of less than 0.05 was considered to be statistically significant. Binary logistic regression analysis was used to determine the relationship between the variables and seroreactivity for anti WNV. Odds ratios and 95% confidence intervals (CI) were calculated for univariate association of various demographic risks correlating with antibodies to WNV. All variables or confounders with statistically significant association in univariate analysis were entered into a 3 steps multivariable logistic regression model, in order to assess the impact of the separate risk factors on the dependent variable. In each step non-significant variable was taken out from the model and calculations were continued after.

## Results

**Fig 2:**
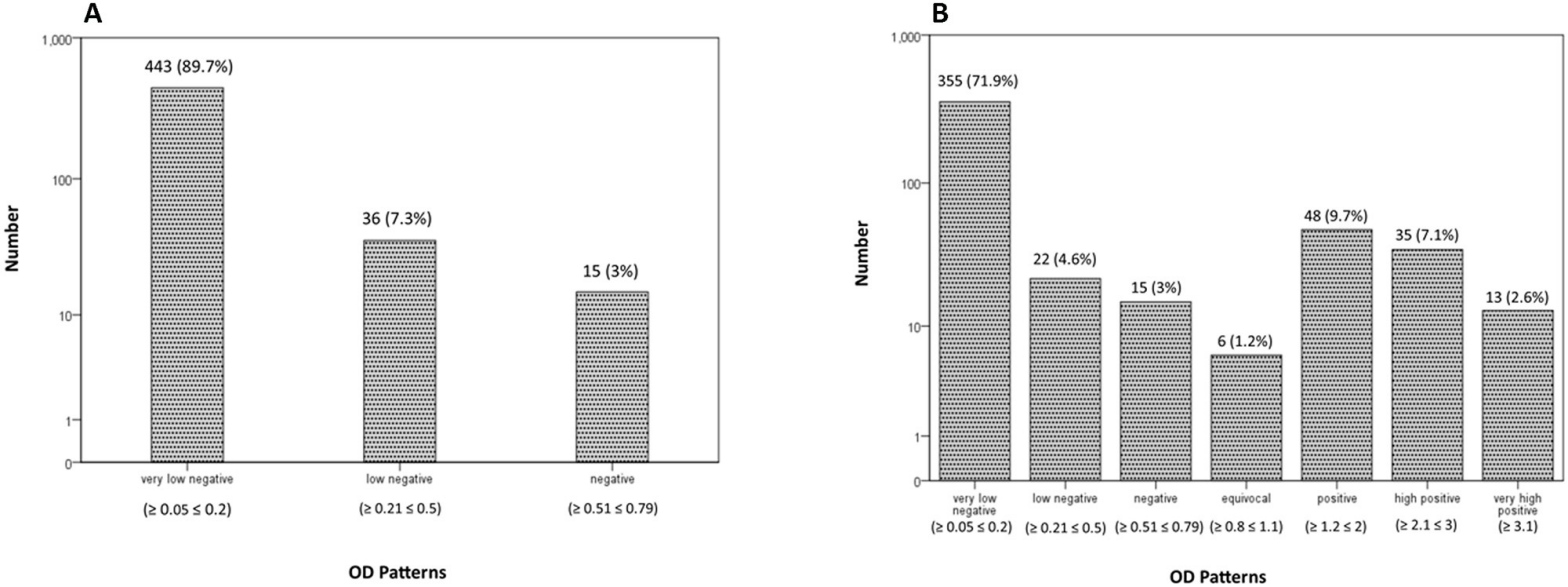
Distribution pattern of ZIKV and WNV IgG OD index. ELISA OD ratios were grouped into seven levels and have been shown for ZIKV (2A) and WNV (2B).

A total of 494 serum samples were tested for the presence of ZIKV and WNV IgG antibodies using ELISA assay. Results showed that 102 (20.6%) samples were reactive (equivocal or positive) for WNV IgG antibodies in the ELISA test, of these 102 samples, 96 were also found positive by IIFT. These 6 IIFT negative serum samples were seroreactive by ELISA (4 were positive and 2 had equivocal results). All serum samples were negative for ZIKV IgG. Pattern of corrected ELISA OD levels values for ZIKV and WNV ELISA tests are shown in Fig 2. The age range of participants was between 1 and 86 years old with a mean age of 33.89, SD = 15.6 years (95% C.I. of mean: 32.5–35.3), with Skewness and Kurtosis of 0.896 and 0.517 respectively. Distribution of study participants by demographic characteristics is shown in Table 1.

**Table 1:** Study populations’ demographic characteristics.

Characteristic	Total Count (n = 494)	Percent (%)
**Age (years)**		
0–15	31	6.3
16–25	128	25.9
26–35	153	31
36–45	78	15.8
46–55	45	9.1
56+	59	11.9
**Gender**		
Female	377	76.3
Male	117	23.7
**Residential area**		
Bandar Khamir	124	25.1
Jask	120	24.3
Bandar Abbas	125	25.3
Bashagard	125	25.3
**Resident type**		
Urban	250	50.6
Rural	244	49.4
**Skin type**		
Type I/II	107	21.7
Type III/IV	334	67.6
Type V/VI	53	10.7
**Occupation**		
Child/student	95	19.2
House wife	288	58.3
Office employee	36	7.3
Freelancer	41	8.3
Fisherman/Sailor	14	2.8
Worker	15	3
Retiree	5	1
**Travelling history**		
No	457	92.5
Yes	37	7.5

According to univariate logistic regression WNV seroprevalenceis significantly associated with age (45+ vs. 1–25; OR = 4.1, 95% C.I.: 2.2–7.4), gender (male vs female; OR = 2, 95% C.I.: 1.2–3.2), residential areas (Bashagard and Bandar Abbas vs. Bandar Khamir; OR = 2.2, 95% C.I.: 1.1–4.2 and OR = 2, 95% C.I.: 1–3.8 respectively), occupation (mostly outdoor vs. mostly indoor; OR = 3.7, 95% C.I.: 1.8–7.7), and skin type (type I/II vs. type III/IV, type V/VI; OR = 3.8, 95% C.I.: 1.6–9.3 and OR = 2.9, 95% C.I.: 1.4–5.8 respectively) (Table 2).

**Table 2:** West Nile virus IgG seroprevalence and univariate analysis by age, gender, residential area, resident type, skin type, occupation and travelling history to outside of country.

Characteristic	Total count (n = 102)	Percent (%)	OR	95%CI	*P*-value
**Age (years)**					
0–25	22	13.8	Ref.		
26–45	39	16.9	1.3	0.7–2.2	0.416
+45	41	39.4	4.1	2.2–7.4	0.000
**Gender**					
Female	67	17.8	Ref.		
Male	35	29.9	2.0	1.2–3.2	0.005
**Residential area**					
Bandar Khamir	17	13.7	Ref.		
Jask	23	19.2	1.5	0.7–3.0	0.252
Bandar Abbas	30	24	2.0	1.0–3.8	0.040
Bashagard	32	25.6	2.2	1.1–4.1	0.020
**Resident type**					
Urban	43	17.2	Ref.		
Rural	59	24.2	1.5	1.0–2.4	0.056
**Skin type**					
Type I/II	10	9.3	Ref.		
Type III/IV	77	23.1	2.9	1.4–5.8	0.003
Type V/VI	15	28.3	3.8	1.6–9.3	0.003
**Occupation**					
Mostly indoor (Child/student/House wife)	67	17.5	Ref.		
Usually indoor (Office employee/ Freelancer)	20	26	1.7	0.9–2.9	0.085
Mostly outdoor (Fisherman/Sailor/ Worker/Retiree)	15	44.1	3.7	1.8–7.7	0.000
**Travelling history**					
No	96	21	Ref.		
Yes	6	16.2	0.7	0.3–1.8	0.490

By multivariable logistic analysis, only age (45+ vs. 1–25; OR = 3.4, 95% C.I.: 1.8–6.3), occupation (mostly outdoor vs. mostly indoor; OR = 2.4, 95% C.I.: 1.1–5.2), and skin type (type I/II vs. type III/IV, type V/VI; OR = 4.3, 95% C.I.: 1.7–10.8 and OR = 2.7, 95% C.I.: 1.3–5.5 respectively) showed significant association with WNV seroreactivity (The degrees of freedom for the model were 10, 8, and 6 for steps 1 to 3 of regression analysis, respectively, Table 3).

**Table 3:** Results of multivariable logistic regression analysis for the assessment of factors associated with WNV seroreactivity.

**Step 1**					
**Characteristic**	**Total count (n = 102)**	**Percent (%)**	**OR**	**95%CI**	***P*-value**
**Age (years)**					
0–25	22	13.8	Ref.		
26–45	39	16.9	1.0	0.5–1.8	0.960
+45	41	39.4	3.3	1.7–6.3	0.001
**Gender**					
Female	67	17.8	Ref.		
Male	35	29.9	1.0	0.4–2.6	0.937
**Residential area**					
Bandar Khamir	17	13.7	Ref.		
Jask	23	19.2	1.4	0.6–2.9	0.417
Bandar Abbas	30	24	1.5	0.7–2.9	0.302
Bashagard	32	25.6	1.8	0.9–3.6	0.090
**Skin type**					
Type I/II	10	9.3	Ref.		
Type III/IV	77	23.1	2.6	1.3–5.4	0.010
Type V/VI	15	28.3	4.3	1.7–11.1	0.002
**Occupation**					
Mostly indoor (Child/student/House wife)	67	17.5	Ref.		
Often indoor (Office employee/ Freelancer)	20	26	1.6	0.6–4.1	0.322
Mostly outdoor (Fisherman/Sailor/ Worker/ Retiree)	15	44.1	2.3	0.7–7.7	0.171
**Step 2**					
**Characteristic**	**Total count (n = 102)**	**Percent (%)**	**OR**	**95%CI**	***P*-value**
**Age (years)**					
0–25	22	13.8	Ref.		
26–45	39	16.9	1.0	0.5–1.8	0.946
+45	41	39.4	3.2	1.7–6.3	0.000
**Residential area**					
Bandar Khamir	17	13.7	Ref.		
Jask	23	19.2	1.4	0.6–2.9	0.419
Bandar Abbas	30	24	1.5	0.7–2.9	0.302
Bashagard	32	25.6	1.8	0.9–3.6	0.089
**Skin type**					
Type I/II	10	9.3	Ref.		
Type III/IV	77	23.1	2.6	1.2–5.4	0.010
Type V/VI	15	28.3	4.3	1.7–11.1	0.002
**Occupation**					
Mostly indoor (Child/student/House wife)	67	17.5	Ref.		
Often indoor(Office employee/ Freelancer)	20	26	1.6	0.9–3.1	0.120
Mostly outdoor(Fisherman/Sailor/ Worker/ Retiree)	15	44.1	2.4	1.1–5.4	0.034
**Step 3**					
**Characteristic**	**Total count (n = 102)**	**Percent (%)**	**OR**	**95%CI**	***P*-value**
**Age (years)**					
0–25	22	13.8	Ref.		
26–45	39	16.9	1.0	0.6–1.8	0.994
+45	41	39.4	3.4	1.8–6.3	0.000
**Skin type**					
Type I/II	10	9.3	Ref.		
Type III/IV	77	23.1	2.7	1.3–5.5	0.008
Type V/VI	15	28.3	4.3	1.7–10.8	0.002
**Occupation**					
Mostly indoor (Child/student/House wife)	67	17.5	Ref.		
Often indoor (Office employee/ Freelancer)	20	26	1.7	0.9–3.2	0.078
Mostly outdoor (Fisherman/Sailor/ Worker/ Retiree)	15	44.1	2.4	1.1–5.2	0.034

Among 2,015 mosquitoes (995 females and 1020 males) belonging to 5 genera and 14 species, were screened for ZIKV and WNV infections (Table 4, S1 Table). ZIKV RNA was not detected in any mosquitoes’ pools. Three of *Cx*. *pipiens* complex pools were positive for WNV RNA. First and second pools consisted mainly of females collected in the districts of Eidar and Sardasht, county of Bashagard; while the third pool had four females collected in the Khargo district of Bandar Abbas county. All WNV ELISA positive serum samples were negative for WNV RNA.

**Table 4:** Species and numbers of collected mosquitoes in Hormozgan, southern Iran.

Mosquito species	County				Number of pools	Number of female (%)	Total count (%)
	Bandar Abbas	Bandar Khamir	Jask	Bashagard			
*Culexpipiens* complex	554	59	40	180	53	415 (20.59)	833 (41.33)
*Anopheles stephensi*	141	11	4	20	14	90 (4.47)	176 (8.73)
*Culex quinquefasciatus*	293	20	45	-	23	173 (8.59)	358 (17.76)
*Culex tritaeniorhynchus*	60	-	20	-	8	33 (1.64)	80 (4)
*Anopheles dthali*	-	2	-	11	3	12 (0.59)	13 (0.65)
*Anopheles fluviatilis*	104	-	8	-	6	59 (2.93)	112 (5.55)
*Culex mimeticus*	5	-	-	5	4	3 (0.15)	10 (0.5)
*Culex laticinctus* Edwards	-	-	-	35	4	15 (0.74)	35 (1.73)
*Aedescaspius*	157	55	-	-	8	98 (4.86)	212 (10.52)
*Aedesvexans*	60	3	-	-	4	32 (1.59)	63 (3.12)
*Culiseta longiareolata*	45	2	-	-	3	27 (1.34)	47 (2.33)
*Culex perexiguus*	8	-	-	-	2	5 (0.25)	8 (0.4)
*Culex theileri*	28	-	-	-	2	8 (0.40)	28 (1.38)
*Uranotaenia unguiculata* Edwards	40	-	-	-	2	25 (1.24)	40 (2)
**Total count (%)**	1495 (74.2)	152 (7.55)	117 (5.8)	251 (12.45)	136	995 (49.38)	2015 (100)

## Discussion

In our study ZIKV serology was negative for all individuals, indeed there is no evidence for past or recent infections with ZIKV in southern areas of Iran. Moreover, two major ZIKV mosquito vectors, *Ae*. *Aegypti* and *Ae*. *Albopictous*, were not among the 5 genera and 14 species of our collected mosquitoes, even though, recent reports have shown the potential for the presence of *Ae*. *albopictous* in Iran [21, 33]. In addition, all mosquitoes’ pools were found negative for ZIKV RNA using rt-RT PCR. Despite the description of ZIKV infection in our neighboring country Pakistan [22] we could not find any evidence of ZIKV infection in the area. The global climate changes severely affecting southern Iran will increase the invasion of ZIKV major vectors and facilitate their adaptation to this new environment. Moreover, the risk of accidental import of ZIKV vectors at the major commercial ports in southern Iran should be also considered. Therefore, establishment of a continuous mosquito surveillance system and controlling of the vector’s population at their mass gathering points seems to be necessary.

In this study we estimated a seroprevalence of 20.6% in WNV ELISA IgG among the studied population. In addition, WNV RNAs were detected in three *Cx*. *Pipiens* pools that were collected from Bashagard and Bandar Abbas. Molecular evidence of WNV in mosquitoes accompanied to high IgG seroprevalence in the present study, suggests high WNV circulation in southern Iran. Previous studies performed in some provinces of Iran have shown seropositivity to WNV in humans (ranging from 1.3% to 17.96%), equine (ranging from 2.8% to 23.7%) and birds (15%); however, they did not identify any possible vectors [23, 24,34, 35]. An earlier study in northwest Iran showed the presence of WNV genome in *Ae*. *caspius* [36]. Based on the present results, it is highly possible that WNV outbreaks had occurred in the past; although, there is no previous documentation supporting this idea. Despite the existence and circulation of WNV, no clinical cases have been described in Iran, so far.

Previous reports from around the world demonstrated that *Ae*. *Aegypti* and *Ae*. *albopictus* mosquitoes possess the ability to transmit ZIKV naturally or experimentally, but there is controversies about the ability of ZIKV transmission by other mosquitospecies. Of the collected mosquitoes in the present study 358 (17.67%) and 63 (3.12%) were *Cx*. *quinquefasciatus* and *Ae*. *vexans* respectively. It is unclear whether these endemic mosquitoes could serve as vectors to spread ZIKV in Iran or not. Different studies including those of Diallo et al., [10]; Guedes et al., [12], and Elizondo-Quiroga et al. [13] indicate that ZIKV has in fact been isolated from *Cx*. *quinquefasciatus* and *Ae*. *vexans* in nature; however, other studies have shown that most *Culex spp*. are not able to transmit the virus under laboratory conditions [37, 38]. Therefore, vector competency studies should be conducted on these endemic mosquito vectors in order to determine the possible ability of these mosquito species to transmit ZIKV.

Some studies showed that serologic evaluation of ZIKV infection is affected by cross-reactivity between antibodies of other flaviviruses mostly dengue viruses, namely in the world endemic areas. Serological and PCR studies have confirmed the presence of dengue virus infections in Iran, but most of them described the infection in association with travelling to hyper endemic areas. Indeed, travelers may play an important role in the epidemiology of dengue virus infections in Iran [39, 40]. In recent studies, it has been demonstrated that the ZIKV Euroimmun ELISA is specific and reliable when compared with other standard serologic methods [41, 42]. Moreover, we also did not detect any reactivity near or above threshold of borderline or positive zone for ZIKV antibodies.

To determine the presence of antibodies against WNV we used two different serological tests, ELISA and IIF. For the most part the results were in agreement (from 102 ELISA reactive samples 96 also showed positive signal by IIF test). Our results in univariate logistic regression showed that, seroprevalence of WNV is higher in males. Indeed, people’s lifestylein the study region is traditional, with most men doing outdoor jobs while most married women stay at home doing housekeeping work and raising children. Therefore, men are more exposed to potential mosquito bites and getting infected because they spend most of the daytime outdoors. Recent studies have been shown a relationship between WNV prevalence and gender [43, 44]. Consistent with our results, there are no significant differences between living in rural or urban areas, because there is no lifestyle disparities between these two populations in this region of the country. Based on the potential of being exposed to the WNV vectors, we categorized the population into three large job groups; first, those who mostly stay indoors, second, those who usually stay indoors and third, those who mostly stay outdoors. As the results show, people with outdoors jobs have significantly higher seroprevalence of WNV in comparison with those who mostly or usually do indoor activities. Similar results have been observed in other countries [45].

Our study shows a significant association of WNV seroreactivity with increase in age. The observed rate was four fold higher in the people who are 45 or older compared to those who are below 25. Impact of age and WNV seroreactivity is consistent with most previous seroprevalence studies in other countries [43, 46], and may be related to higher probability of experiencing and being exposed to WNV among older individuals during their lifetimes.

One important finding of the study is the significant differences in WNV seroprevalence among people with different skin types. According to our data, the risk of WNV infections among individuals who have type III/IV or V/VI skin complexion are three to four times higher than individuals with the skin type I/II, respectively. This result may be related to non-protected exposure of the skin to the sun and to uncovered environment.

In univariate analysis a significant relationship was found between the residential area and WNV seroreactivity. People residing in the Bashagard and Bandar Abbas had the highest seroprevalence of WNV antibodies when compared with those living in Jask and Khamir. This finding may be related to a mild difference in environmental factors including altitude, distance to the nearest wetland area and normalized difference vegetation index (NDVI) of the Bashagard and Bandar Abbas counties in comparison with the Jask and Khamir counties, which may have an effect on the ecology of the vectors. Similar effects of these environmental factors have been observed in previous studies [47].

A limitation of the present study is that we looked for the presence of WNV and ZIKV RNA in the sera samples. However, some reports suggest that the copy numbers of WNV and ZIKV genome is higher in whole blood or urine in comparison to sera samples [48]; which may explain in part why no RNA was detected in the sera samples.

In conclusion, our results provide the evidence of the establishment of WNV in southern Iran with a seroprevalence of 20.6% and no proof of ZIKV presence in mosquito vectors or serology markers in humans. Moreover, people who mainly work at open areas and the elderly are at higher risk of getting infected by WNV. In these study areas even though some people showed positive serum results for WNV infection, there were no recorded human WNV cases. As elucidated before, most people in southern Iran have traditional lifestyles where some moderate fever or temporary rashes do not make them visit medical facilities. Other important factors that might obscure not only WNV cases but also other mosquito-borne infections in these studied regions and even in other parts of Iran are the lack of an organized arbovirus surveillance system and active case finding and reporting strategies. These results generated new knowledge that is critical to better understand the epidemiology of the infection and the ecology of the vectors in the region. Furthermore, these findings will help to establish the basis for developing a better surveillance system with improved active case finding and reporting strategies for these arboviruses.

## Supporting information

S1 Table Geographical, environmental properties and composition of mosquitoes’ pools collected in Hormozgan province, southern Iran. (DOC)
