# Supplementary material for: Widespread circulation of West Nile virus, but not Zika virus in southern Iran
Source: PLoS Negl Trop Dis. 2018 Dec 17;12(12):e0007022. doi: 10.1371/journal.pntd.0007022 (PMC6312345; doi:10.1371/journal.pntd.0007022)
Supplement: S1 Table — (DOC) [file pntd.0007022.s001.doc]

| **GPS Coordinates (latitude and longitude)** | **Moisture %** | **Temperature(°C)** | **County** | **Location** | **Date of Catching** | **Number of Blood Feeding Mosquitoes** | **Gender** | **No. of Mosquitoes in Tube** | **Species** | **Pool's Number** |
| --- | --- | --- | --- | --- | --- | --- | --- | --- | --- | --- |
| N: 26°45'41.9''  E: 57°90'51.5'' | 58 | 23 | Bashagard | Human living area (indoor) | Mar. 2016 | 0 | Male | 10 | *Culexpipiens* complex | 1 |
| N: 26°45'26.0''  E: 57°90'47.2'' | 58 | 23 | Bashagard | Human living area (indoor) | Mar. 2016 | 0 | Male | 10 | *Culexpipiens* complex | 2 |
| N: 26°45'86.1''  E: 57°90'38.2'' | 58 | 23 | Bashagard | Human living area (indoor) | Mar. 2016 | 0 | Male | 10 | *Culexpipiens* complex | 3 |
| N: 26°45'84.2''  E: 57°90'41.9'' | 58 | 23 | Bashagard | Human living area (indoor) | Mar. 2016 | 0 | Male | 10 | *Culexpipiens* complex | 4 |
| N: 26°45'40.5''  E: 57°90'56.8'' | 58 | 23 | Bashagard | Animal living area (outdoor) | Mar. 2016 | 0 | Male | 10 | *Culexpipiens* complex | 5 |
| N: 26°45'98.0''  E: 57°90'61.0'' | 58 | 23 | Bashagard | Animal living area (outdoor) | Mar. 2016 | 0 | Male | 10 | *Culexpipiens* complex | 6 |
| N: 26°45'87.8''  E: 57°90'47.1'' | 58 | 23 | Bashagard | Animal living area (outdoor) | Mar. 2016 | 0 | Male | 10 | *Culexpipiens* complex | 7 |
| N: 26°45'82.4''  E: 57°90'35.8'' | 58 | 23 | Bashagard | Animal living area (outdoor) | Mar. 2016 | 0 | Male | 8 | *Culexpipiens* complex | 8 |
| N: 26°46'75.7''  E: 57°90'49.8'' | 58 | 22 | Bashagard | Human living area (indoor) | Mar. 2016 | 0 | Male | 10 | *Culexpipiens* complex | 9 |
| N: 26°45'87.2''  E: 57.90351° | 58 | 22 | Bashagard | Human living area (indoor) | Mar. 2016 | 0 | Male | 5 | *Culexpipiens* complex | 10 |
| N: 26°59'38.2''  E: 57°89'80.6'' | 74 | 27 | Bashagard | Human living area (indoor) | Sep. 2016 | 0 | Male | 10 | *Culexpipiens* complex | 11 |
| N: 26°59'38.2''  E: 57°89'80.6'' | 74 | 27 | Bashagard | Human living area (indoor) | Sep. 2016 | 2 | Female | 2 | *Culexpipiens* complex | 12 |
| N: 26°30'44.8''  E: 57°66'97.8'' | 74 | 31 | Bashagard | Human living area (indoor) | Sep. 2016 | 0 | Male | 2 | *Culexpipiens* complex | 13 |
| N: 26°30'44.8''  E: 57°66'97.8'' | 74 | 31 | Bashagard | Human living area (indoor) | Sep. 2016 | 0 | Female | 2 | *Culexpipiens* complex | 14 |
| N: 25°78'96.9''  E: 58°17'19.8'' | 69 | 30 | Jask | Human living area (indoor) | Sep. 2016 | 0 | Male | 2 | [*Anopheles stephensi*](https://en.wikipedia.org/wiki/Anopheles_stephensi) | 15 |
| N: 25°78'96.9''  E: 58°17'19.8'' | 69 | 30 | Jask | Human living area (indoor) | Sep. 2016 | 1 | Female | 2 | [*Anopheles stephens*i](https://en.wikipedia.org/wiki/Anopheles_stephensi) | 16 |
| N: 25°71'94.9''  E: 58°19'57.1'' | 64 | 32 | Jask | Human living area (indoor) | Sep. 2016 | 0 | Male | 7 | [*Culexquinquefasciatus*](https://www.google.com/url?sa=t&rct=j&q=&esrc=s&source=web&cd=1&cad=rja&uact=8&ved=0ahUKEwj70rjt_r3ZAhUEKuwKHRuFCssQFggqMAA&url=https%3A%2F%2Fen.wikipedia.org%2Fwiki%2FCulex_quinquefasciatus&usg=AOvVaw39EYPbl6oFUCKPSf86_ABm) | 17 |
| N: 25°71'94.9''  E: 58°19'57.1'' | 64 | 32 | Jask | Human living area (indoor) | Sep. 2016 | 0 | Female | 1 | [*Culexquinquefasciatus*](https://www.google.com/url?sa=t&rct=j&q=&esrc=s&source=web&cd=1&cad=rja&uact=8&ved=0ahUKEwj70rjt_r3ZAhUEKuwKHRuFCssQFggqMAA&url=https%3A%2F%2Fen.wikipedia.org%2Fwiki%2FCulex_quinquefasciatus&usg=AOvVaw39EYPbl6oFUCKPSf86_ABm) | 18 |
| N: 25°82'65.4''  E: 58°17'17.7'' | 62 | 31 | Jask | Human living area (indoor) | Sep. 2016 | 0 | Male | 19 | [*Culextritaeniorhynchus*](https://www.google.com/url?sa=t&rct=j&q=&esrc=s&source=web&cd=1&cad=rja&uact=8&ved=0ahUKEwiNlM-j_73ZAhUssKQKHdJGDfsQFggqMAA&url=https%3A%2F%2Fen.wikipedia.org%2Fwiki%2FCulex_tritaeniorhynchus&usg=AOvVaw2aeyiIubUP8TQbXf16a5na) | 19 |
| N: 25°82'65.4''  E: 58°17'17.7'' | 62 | 31 | Jask | Human living area (indoor) | Sep. 2016 | 0 | Female | 1 | [*Culextritaeniorhynchus*](https://www.google.com/url?sa=t&rct=j&q=&esrc=s&source=web&cd=1&cad=rja&uact=8&ved=0ahUKEwiNlM-j_73ZAhUssKQKHdJGDfsQFggqMAA&url=https%3A%2F%2Fen.wikipedia.org%2Fwiki%2FCulex_tritaeniorhynchus&usg=AOvVaw2aeyiIubUP8TQbXf16a5na) | 20 |
| N: 26°75'38.9''  E: 57°83'89.4'' | 67 | 26 | Bashagard | Human living area (indoor) | Sep. 2016 | 0 | Male | 5 | *Culexpipiens*complex | 21 |
| N: 26°75'38.9''  E: 57°83'89.4'' | 67 | 26 | Bashagard | Human living area (indoor) | Sep. 2016 | 0 | Female | 2 | *Culexpipiens*complex | 22 |
| N: 26°75'38.9''  E: 57°83'89.4'' | 67 | 26 | Bashagard | Animal living area (outdoor) | Sep. 2016 | 0 | Male | 10 | [*Anopheles stephensi*](https://en.wikipedia.org/wiki/Anopheles_stephensi) | 23 |
| N: 26°75'38.9''  E: 57°83'89.4'' | 67 | 26 | Bashagard | Animal living area (outdoor) | Sep. 2016 | 3 | Female | 10 | [*Anopheles stephensi*](https://en.wikipedia.org/wiki/Anopheles_stephensi) | 24 |
| N: 26°46'75.7''  E: 57°90'49.8'' | 62 | 28 | Bashagard | Human living area (indoor) | Oct. 2016 | 0 | Male | 6 | *Culexpipiens*complex | 25 |
| N: 26°46'75.7''  E: 57°90'49.8'' | 62 | 28 | Bashagard | Human living area (indoor) | Oct. 2016 | 0 | Female | 1 | *Culexpipiens*complex | 26 |
| N: 26°67'85.4''  E: 57°90'55.9'' | 40 | 26 | Bashagard | Animal living area (outdoor) | Oct. 2016 | 0 | Male | 1 | *Anopheles dthali* | 27 |
| N: 26°67'85.4''  E: 57°90'55.9'' | 40 | 26 | Bashagard | Animal living area (outdoor) | Oct. 2016 | 2 | Female | 10 | *Anopheles dthali* | 28 |
| N: 26°67'85.4''  E: 57°90'55.9'' | 38 | 30 | Bashagard | Human living area (indoor) | Oct. 2016 | 0 | Male | 7 | *Culexpipiens*complex | 29 |
| N: 26°67'85.4''  E: 57°90'55.9'' | 38 | 30 | Bashagard | Human living area (indoor) | Oct. 2016 | 2 | Female | 10 | *Culexpipiens*complex | 30+ |
| N: 26°57'29''  E: 55°36'26'' | 60 | 32 | Bandar Khmair | Animal living area (indoor) | Oct. 2016 | 1 | Female | 2 | [*Anopheles stephensi*](https://en.wikipedia.org/wiki/Anopheles_stephensi) | 31 |
| N: 26°57'29''  E: 55°36'26'' | 60 | 32 | Bandar Khmair | Human living area (indoor) | Oct. 2016 | 1 | Female | 3 | *Culexpipiens* complex | 32 |
| N: 26°57'29''  E: 55°36'26'' | 60 | 32 | Bandar Khmair | Human living area (indoor) | Oct. 2016 | 2 | Female | 3 | [*Culexquinquefasciatus*](https://www.google.com/url?sa=t&rct=j&q=&esrc=s&source=web&cd=1&cad=rja&uact=8&ved=0ahUKEwj70rjt_r3ZAhUEKuwKHRuFCssQFggqMAA&url=https%3A%2F%2Fen.wikipedia.org%2Fwiki%2FCulex_quinquefasciatus&usg=AOvVaw39EYPbl6oFUCKPSf86_ABm) | 33 |
| N: 26°57'29''  E: 55°36'26'' | 60 | 32 | Bandar Khmair | Human living area (indoor) | Oct. 2016 | 0 | Male | 2 | *Culexpipiens* complex | 34 |
| N: 26°57'29''  E: 55°36'26'' | 60 | 32 | Bandar Khmair | Human living area (indoor) | Oct. 2016 | 0 | Male | 1 | [*Culexquinquefasciatus*](https://www.google.com/url?sa=t&rct=j&q=&esrc=s&source=web&cd=1&cad=rja&uact=8&ved=0ahUKEwj70rjt_r3ZAhUEKuwKHRuFCssQFggqMAA&url=https%3A%2F%2Fen.wikipedia.org%2Fwiki%2FCulex_quinquefasciatus&usg=AOvVaw39EYPbl6oFUCKPSf86_ABm) | 35 |
| N: 27°18'57''  E: 56°18'43'' | 63 | 34 | Bandar Abbas | Human living area (indoor) | Oct. 2016 | 1 | Female | 5 | *Culexpipiens* complex | 36 |
| N: 27°18'57''  E: 56°18'43'' | 63 | 34 | Bandar Abbas | Human living area (indoor) | Oct. 2016 | 0 | Male | 3 | *Culexpipiens* complex | 37 |
| N: 27°18'57''  E: 56°18'43'' | 63 | 34 | Bandar Abbas | Animal living area (indoor) | Oct. 2016 | 4 | Female | 4 | [*Anopheles stephensi*](https://en.wikipedia.org/wiki/Anopheles_stephensi) | 38 |
| N: 27°18'57''  E: 56°18'43'' | 63 | 34 | Bandar Abbas | Animal living area (indoor) | Oct. 2016 | 0 | Male | 2 | [*Anopheles stephensi*](https://en.wikipedia.org/wiki/Anopheles_stephensi) | 39 |
| N: 27°18'57''  E: 56°18'43'' | 63 | 34 | Bandar Abbas | Animal living area (indoor) | Oct. 2016 | 1 | Female | 3 | *Anopheles fluviatilis* | 40 |
| N: 27°18'57''  E: 56°18'43'' | 63 | 34 | Bandar Abbas | Animal living area (indoor) | Oct. 2016 | 0 | Male | 1 | *Anopheles fluviatilis* | 41 |
| N: 25°79'02.6''  E: 58°17'68.0'' | 65 | 30 | Jask | Human living area (indoor) | Oct. 2016 | 0 | Male | 10 | [*Culexquinquefasciatus*](https://www.google.com/url?sa=t&rct=j&q=&esrc=s&source=web&cd=1&cad=rja&uact=8&ved=0ahUKEwj70rjt_r3ZAhUEKuwKHRuFCssQFggqMAA&url=https%3A%2F%2Fen.wikipedia.org%2Fwiki%2FCulex_quinquefasciatus&usg=AOvVaw39EYPbl6oFUCKPSf86_ABm) | 42 |
| N: 25°79'02.6''  E: 58°17'68.0'' | 65 | 30 | Jask | Human living area (indoor) | Oct. 2016 | 4 | Female | 10 | [*Culexquinquefasciatus*](https://www.google.com/url?sa=t&rct=j&q=&esrc=s&source=web&cd=1&cad=rja&uact=8&ved=0ahUKEwj70rjt_r3ZAhUEKuwKHRuFCssQFggqMAA&url=https%3A%2F%2Fen.wikipedia.org%2Fwiki%2FCulex_quinquefasciatus&usg=AOvVaw39EYPbl6oFUCKPSf86_ABm) | 43 |
| N: 25°79'33.6''  E: 58°17'27.2'' | 56 | 30 | Jask | Shelter pit | Oct. 2016 | 0 | Male | 10 | [*Culexquinquefasciatus*](https://www.google.com/url?sa=t&rct=j&q=&esrc=s&source=web&cd=1&cad=rja&uact=8&ved=0ahUKEwj70rjt_r3ZAhUEKuwKHRuFCssQFggqMAA&url=https%3A%2F%2Fen.wikipedia.org%2Fwiki%2FCulex_quinquefasciatus&usg=AOvVaw39EYPbl6oFUCKPSf86_ABm) | 44 |
| N: 25°79'33.6''  E: 58°17'27.2'' | 56 | 30 | Jask | Shelter pit | Oct. 2016 | 1 | Female | 7 | [*Culexquinquefasciatus*](https://www.google.com/url?sa=t&rct=j&q=&esrc=s&source=web&cd=1&cad=rja&uact=8&ved=0ahUKEwj70rjt_r3ZAhUEKuwKHRuFCssQFggqMAA&url=https%3A%2F%2Fen.wikipedia.org%2Fwiki%2FCulex_quinquefasciatus&usg=AOvVaw39EYPbl6oFUCKPSf86_ABm) | 45 |
| N: 25°75'24.0''  E: 58°44'58.7'' | 53 | 32 | Jask | Animal living area (outdoor) | Oct. 2016 | 0 | Male | 2 | *Anopheles fluviatilis* | 46 |
| N: 25°75'24.0''  E: 58°44'58.7'' | 53 | 32 | Jask | Animal living area (outdoor) | Oct. 2016 | 2 | Female | 6 | *Anopheles fluviatilis* | 47 |
| N: 26°45'87.3''  E: 57°90'34.2'' | 29 | 17 | Bashagard | Human living area (indoor) | Nov. 2016 | 0 | Male | 3 | *Culexmimeticus* | 48 |
| N: 26°45'87.3''  E: 57°90'34.2'' | 29 | 17 | Bashagard | Human living area (indoor) | Nov. 2016 | 0 | Female | 2 | *Culexmimeticus* | 49 |
| N: 26°45'87.3''  E: 57°90'34.2'' | 29 | 17 | Bashagard | Animal living area (outdoor) | Nov. 2016 | 0 | Male | 10 | *Culexpipiens*complex | 50 |
| N: 26°45'87.3''  E: 57°90'34.2'' | 29 | 17 | Bashagard | Animal living area (outdoor) | Nov. 2016 | 1 | Female | 10 | *Culexpipiens*complex | 51+ |
| N: 26°45'58.3''  E: 57°75'15.7'' | 33 | 16 | Bashagard | Human living area (indoor) | Nov. 2016 | 0 | Male | 10 | [*Culexlaticinctus* Edwards](https://www.google.com/url?sa=t&rct=j&q=&esrc=s&source=web&cd=6&cad=rja&uact=8&ved=0ahUKEwjB0sLsgr7ZAhXGJ1AKHY8pAt4QFgg1MAU&url=https%3A%2F%2Fwww.cabi.org%2Fisc%2Fabstract%2F19950508848&usg=AOvVaw3F9FVQZ6G-7w5h9xEClSCm) | 52 |
| N: 26°45'58.3''  E: 57°75'15.7'' | 33 | 16 | Bashagard | Human living area (indoor) | Nov. 2016 | 1 | Female | 7 | [*Culexlaticinctus* Edwards](https://www.google.com/url?sa=t&rct=j&q=&esrc=s&source=web&cd=6&cad=rja&uact=8&ved=0ahUKEwjB0sLsgr7ZAhXGJ1AKHY8pAt4QFgg1MAU&url=https%3A%2F%2Fwww.cabi.org%2Fisc%2Fabstract%2F19950508848&usg=AOvVaw3F9FVQZ6G-7w5h9xEClSCm) | 53 |
| N: 26°45'58.3''  E: 57°75'15.7'' | 31 | 15 | Bashagard | Human living area (indoor) | Nov. 2016 | 0 | Male | 10 | [*Culexlaticinctus* Edwards](https://www.google.com/url?sa=t&rct=j&q=&esrc=s&source=web&cd=6&cad=rja&uact=8&ved=0ahUKEwjB0sLsgr7ZAhXGJ1AKHY8pAt4QFgg1MAU&url=https%3A%2F%2Fwww.cabi.org%2Fisc%2Fabstract%2F19950508848&usg=AOvVaw3F9FVQZ6G-7w5h9xEClSCm) | 54 |
| N: 26°45'58.3''  E: 57°75'15.7'' | 31 | 15 | Bashagard | Human living area (indoor) | Nov. 2016 | 2 | Female | 8 | [*Culexlaticinctus* Edwards](https://www.google.com/url?sa=t&rct=j&q=&esrc=s&source=web&cd=6&cad=rja&uact=8&ved=0ahUKEwjB0sLsgr7ZAhXGJ1AKHY8pAt4QFgg1MAU&url=https%3A%2F%2Fwww.cabi.org%2Fisc%2Fabstract%2F19950508848&usg=AOvVaw3F9FVQZ6G-7w5h9xEClSCm) | 55 |
| N: 26°45'58.3''  E: 57°75'15.7'' | 30 | 17 | Bashagard | Animal living area (outdoor) | Nov. 2016 | 0 | Male | 10 | *Culexpipiens*complex | 56 |
| N: 26°45'58.3''  E: 57°75'15.7'' | 30 | 17 | Bashagard | Animal living area (outdoor) | Nov. 2016 | 0 | Female | 10 | *Culexpipiens*complex | 57 |
| N: 25°78'90.7''  E: 58°17'37.2'' | 53 | 22 | Jask | Human living area (indoor) | Nov. 2016 | 0 | Male | 10 | *Culexpipiens*complex | 58 |
| N: 25°78'90.7''  E: 58°17'37.2'' | 53 | 22 | Jask | Human living area (indoor) | Nov. 2016 | 3 | Female | 10 | *Culexpipiens*complex | 59 |
| N: 25°78'90.7''  E: 58°17'37.2'' | 53 | 22 | Jask | Human living area (indoor) | Nov. 2016 | 0 | Male | 10 | *Culexpipiens*complex | 60 |
| N: 25°78'90.7''  E: 58°17'37.2'' | 53 | 22 | Jask | Human living area (indoor) | Nov. 2016 | 2 | Female | 10 | *Culexpipiens*complex | 61 |
| N: 25°78'90.7''  E: 58°17'37.2'' | 53 | 22 | Jask | Animal living area (outdoor) | Nov. 2016 | 1 | Female | 5 | [*Culexquinquefasciatus*](https://www.google.com/url?sa=t&rct=j&q=&esrc=s&source=web&cd=1&cad=rja&uact=8&ved=0ahUKEwj70rjt_r3ZAhUEKuwKHRuFCssQFggqMAA&url=https%3A%2F%2Fen.wikipedia.org%2Fwiki%2FCulex_quinquefasciatus&usg=AOvVaw39EYPbl6oFUCKPSf86_ABm) | 62 |
| N: 27°38'24''  E: 55°53'46'' | 65 | 32 | Bandar Abbas | Animal living area (indoor) | Dec. 2016 | 0 | Female | 25 | [*Culextritaeniorhynchus*](https://www.google.com/url?sa=t&rct=j&q=&esrc=s&source=web&cd=1&cad=rja&uact=8&ved=0ahUKEwiNlM-j_73ZAhUssKQKHdJGDfsQFggqMAA&url=https%3A%2F%2Fen.wikipedia.org%2Fwiki%2FCulex_tritaeniorhynchus&usg=AOvVaw2aeyiIubUP8TQbXf16a5na) | 63 |
| N: 27°38'24''  E: 55°53'46'' | 65 | 32 | Bandar Abbas | Animal living area (indoor) | Dec. 2016 | 0 | Male | 25 | [*Culextritaeniorhynchus*](https://www.google.com/url?sa=t&rct=j&q=&esrc=s&source=web&cd=1&cad=rja&uact=8&ved=0ahUKEwiNlM-j_73ZAhUssKQKHdJGDfsQFggqMAA&url=https%3A%2F%2Fen.wikipedia.org%2Fwiki%2FCulex_tritaeniorhynchus&usg=AOvVaw2aeyiIubUP8TQbXf16a5na) | 64 |
| N: 27°33'16''  E: 56°26'51'' | 48 | 25 | Bandar Abbas | Human living area (outdoor) | Dec. 2016 | 20 | Female | 50 | [*Culexquinquefasciatus*](https://www.google.com/url?sa=t&rct=j&q=&esrc=s&source=web&cd=1&cad=rja&uact=8&ved=0ahUKEwj70rjt_r3ZAhUEKuwKHRuFCssQFggqMAA&url=https%3A%2F%2Fen.wikipedia.org%2Fwiki%2FCulex_quinquefasciatus&usg=AOvVaw39EYPbl6oFUCKPSf86_ABm) | 65 |
| N: 27°33'16''  E: 56°26'51'' | 48 | 25 | Bandar Abbas | Human living area (outdoor) | Dec. 2016 | 0 | Male | 50 | [*Culexquinquefasciatus*](https://www.google.com/url?sa=t&rct=j&q=&esrc=s&source=web&cd=1&cad=rja&uact=8&ved=0ahUKEwj70rjt_r3ZAhUEKuwKHRuFCssQFggqMAA&url=https%3A%2F%2Fen.wikipedia.org%2Fwiki%2FCulex_quinquefasciatus&usg=AOvVaw39EYPbl6oFUCKPSf86_ABm) | 66 |
| N: 27°33'16''  E: 56°26'51'' | 48 | 25 | Bandar Abbas | Human living area (outdoor) | Dec. 2016 | 0 | Female | 30 | *Aedesvexans* | 67 |
| N: 27°33'16''  E: 56°26'51'' | 48 | 25 | Bandar Abbas | Human living area (outdoor) | Dec. 2016 | 0 | Male | 30 | *Aedesvexans* | 68 |
| N: 27°38'24''  E: 55°53'46'' | 65 | 32 | Bandar Abbas | Human living area (indoor) | Dec. 2016 | 1 | Female | 6 | *Culexpipiens*complex | 69 |
| N: 27°38'24''  E: 55°53'46'' | 65 | 32 | Bandar Abbas | Human living area (indoor) | Dec. 2016 | 0 | Male | 3 | *Culexpipiens* complex | 70 |
| N: 27°38'24''  E: 55°53'46'' | 65 | 32 | Bandar Abbas | Animal living area (outdoor) | Dec. 2016 | 0 | Female | 1 | *Culexmimeticus* | 71 |
| N: 27°38'24''  E: 55°53'46'' | 65 | 32 | Bandar Abbas | Animal living area (outdoor) | Dec. 2016 | 0 | Male | 4 | *Culexmimeticus* | 72 |
| N: 26°38'29''  E: 55°44'58'' | 65 | 30 | Bandar Khmair | Human living area (indoor) | Dec. 2016 | 2 | Female | 4 | [*Anopheles stephensi*](https://en.wikipedia.org/wiki/Anopheles_stephensi) | 73 |
| N: 26°38'29''  E: 55°44'58'' | 65 | 30 | Bandar Khmair | Human living area (indoor) | Dec. 2016 | 0 | Male | 2 | [*Anopheles stephensi*](https://en.wikipedia.org/wiki/Anopheles_stephensi) | 74 |
| N: 26°38'29''  E: 55°44'58'' | 65 | 30 | Bandar Khmair | Boat | Dec. 2016 | 0 | Female | 3 | *Aedescaspius* | 75 |
| N: 26°38'29''  E: 55°44'58'' | 65 | 30 | Bandar Khmair | Boat | Dec. 2016 | 0 | Male | 2 | *Aedescaspius* | 76 |
| N: 26°38'29''  E: 55°44'58'' | 65 | 30 | Bandar Khmair | Human living area (indoor) | Dec. 2016 | 1 | Female | 5 | *Culexpipiens* complex | 77 |
| N: 26°38'29''  E: 55°44'58'' | 65 | 30 | Bandar Khmair | Human living area (indoor) | Dec. 2016 | 0 | Male | 5 | *Culexpipiens* complex | 78 |
| N: 26°38'29''  E: 55°44'58'' | 65 | 30 | Bandar Khmair | Human living area (indoor) | Dec. 2016 | 2 | Female | 3 | [*Culexquinquefasciatus*](https://www.google.com/url?sa=t&rct=j&q=&esrc=s&source=web&cd=1&cad=rja&uact=8&ved=0ahUKEwj70rjt_r3ZAhUEKuwKHRuFCssQFggqMAA&url=https%3A%2F%2Fen.wikipedia.org%2Fwiki%2FCulex_quinquefasciatus&usg=AOvVaw39EYPbl6oFUCKPSf86_ABm) | 79 |
| N: 26°38'29''  E: 55°44'58'' | 65 | 30 | Bandar Khmair | Human living area (indoor) | Dec. 2016 | 0 | Male | 2 | [*Culexquinquefasciatus*](https://www.google.com/url?sa=t&rct=j&q=&esrc=s&source=web&cd=1&cad=rja&uact=8&ved=0ahUKEwj70rjt_r3ZAhUEKuwKHRuFCssQFggqMAA&url=https%3A%2F%2Fen.wikipedia.org%2Fwiki%2FCulex_quinquefasciatus&usg=AOvVaw39EYPbl6oFUCKPSf86_ABm) | 80 |
| N: 27°38'24''  E: 55°53'46'' | 65 | 32 | Bandar Abbas | Animal living area (outdoor) | Dec. 2016 | 0 | Female | 6 | [*Culextritaeniorhynchus*](https://www.google.com/url?sa=t&rct=j&q=&esrc=s&source=web&cd=1&cad=rja&uact=8&ved=0ahUKEwiNlM-j_73ZAhUssKQKHdJGDfsQFggqMAA&url=https%3A%2F%2Fen.wikipedia.org%2Fwiki%2FCulex_tritaeniorhynchus&usg=AOvVaw2aeyiIubUP8TQbXf16a5na) | 81 |
| N: 27°38'24''  E: 55°53'46'' | 65 | 32 | Bandar Abbas | Animal living area (outdoor) | Dec. 2016 | 0 | Male | 2 | [*Culextritaeniorhynchus*](https://www.google.com/url?sa=t&rct=j&q=&esrc=s&source=web&cd=1&cad=rja&uact=8&ved=0ahUKEwiNlM-j_73ZAhUssKQKHdJGDfsQFggqMAA&url=https%3A%2F%2Fen.wikipedia.org%2Fwiki%2FCulex_tritaeniorhynchus&usg=AOvVaw2aeyiIubUP8TQbXf16a5na) | 82 |
| N: 27°38'24''  E: 55°53'46'' | 65 | 32 | Bandar Abbas | Animal living area (outdoor) | Dec. 2016 | 3 | Female | 5 | *Culexperexiguus* | 83 |
| N: 27°38'24''  E: 55°53'46'' | 65 | 32 | Bandar Abbas | Animal living area (outdoor) | Dec. 2016 | 0 | Male | 3 | *Culexperexiguus* | 84 |
| N: 27°33'16''  E: 56°26'51'' | 48 | 25 | Bandar Abbas | Human living area (indoor) | Dec. 2016 | 4 | Female | 4 | *Culexpipiens* complex | 85+ |
| N: 27°33'16''  E: 56°26'51'' | 48 | 25 | Bandar Abbas | Human living area (indoor) | Dec. 2016 | 0 | Male | 3 | *Culexpipiens* complex | 86 |
| N: 27°33'16''  E: 56°26'51'' | 48 | 25 | Bandar Abbas | Human living area (indoor) | Dec. 2016 | 1 | Female | 1 | [*Culextritaeniorhynchus*](https://www.google.com/url?sa=t&rct=j&q=&esrc=s&source=web&cd=1&cad=rja&uact=8&ved=0ahUKEwiNlM-j_73ZAhUssKQKHdJGDfsQFggqMAA&url=https%3A%2F%2Fen.wikipedia.org%2Fwiki%2FCulex_tritaeniorhynchus&usg=AOvVaw2aeyiIubUP8TQbXf16a5na) | 87 |
| N: 27°33'16''  E: 56°26'51'' | 48 | 25 | Bandar Abbas | Human living area (indoor) | Dec. 2016 | 0 | Male | 1 | [*Culextritaeniorhynchus*](https://www.google.com/url?sa=t&rct=j&q=&esrc=s&source=web&cd=1&cad=rja&uact=8&ved=0ahUKEwiNlM-j_73ZAhUssKQKHdJGDfsQFggqMAA&url=https%3A%2F%2Fen.wikipedia.org%2Fwiki%2FCulex_tritaeniorhynchus&usg=AOvVaw2aeyiIubUP8TQbXf16a5na) | 88 |
| N: 27°33'16''  E: 56°26'51'' | 48 | 25 | Bandar Abbas | Human living area (indoor) | Dec. 2016 | 2 | Female | 2 | [*Culexquinquefasciatus*](https://www.google.com/url?sa=t&rct=j&q=&esrc=s&source=web&cd=1&cad=rja&uact=8&ved=0ahUKEwj70rjt_r3ZAhUEKuwKHRuFCssQFggqMAA&url=https%3A%2F%2Fen.wikipedia.org%2Fwiki%2FCulex_quinquefasciatus&usg=AOvVaw39EYPbl6oFUCKPSf86_ABm) | 89 |
| N: 27°33'16''  E: 56°26'51'' | 48 | 25 | Bandar Abbas | Human living area (indoor) | Dec. 2016 | 0 | Male | 1 | [*Culexquinquefasciatus*](https://www.google.com/url?sa=t&rct=j&q=&esrc=s&source=web&cd=1&cad=rja&uact=8&ved=0ahUKEwj70rjt_r3ZAhUEKuwKHRuFCssQFggqMAA&url=https%3A%2F%2Fen.wikipedia.org%2Fwiki%2FCulex_quinquefasciatus&usg=AOvVaw39EYPbl6oFUCKPSf86_ABm) | 90 |
| N: 27°12'56''  E: 55°87'84'' | 45 | 18 | Bandar Abbas | Human living area (outdoor) | Jan. 2017 | 21 | Female | 40 | *Culexpipiens* complex | 91 |
| N: 27°12'56''  E: 55°87'84'' | 45 | 18 | Bandar Abbas | Human living area (outdoor) | Jan. 2017 | 0 | Male | 25 | *Culexpipiens* complex | 92 |
| N: 27°12'56''  E: 55°87'84'' | 45 | 18 | Bandar Abbas | Human living area (outdoor) | Jan. 2017 | 2 | Female | 15 | [*Culexquinquefasciatus*](https://www.google.com/url?sa=t&rct=j&q=&esrc=s&source=web&cd=1&cad=rja&uact=8&ved=0ahUKEwj70rjt_r3ZAhUEKuwKHRuFCssQFggqMAA&url=https%3A%2F%2Fen.wikipedia.org%2Fwiki%2FCulex_quinquefasciatus&usg=AOvVaw39EYPbl6oFUCKPSf86_ABm) | 93 |
| N: 27°12'56''  E: 55°87'84'' | 45 | 18 | Bandar Abbas | Human living area (outdoor) | Jan. 2017 | 0 | Male | 25 | [*Culexquinquefasciatus*](https://www.google.com/url?sa=t&rct=j&q=&esrc=s&source=web&cd=1&cad=rja&uact=8&ved=0ahUKEwj70rjt_r3ZAhUEKuwKHRuFCssQFggqMAA&url=https%3A%2F%2Fen.wikipedia.org%2Fwiki%2FCulex_quinquefasciatus&usg=AOvVaw39EYPbl6oFUCKPSf86_ABm) | 94 |
| N: 27°38'24''  E: 55°53'46'' | 30 | 22 | Bandar Abbas | Human living area (outdoor) | Jan. 2017 | 4 | Female | 50 | *Culexpipiens* complex | 95 |
| N: 27°38'24''  E: 55°53'46'' | 30 | 22 | Bandar Abbas | Human living area (outdoor) | Jan. 2017 | 0 | Male | 50 | *Culexpipiens* complex | 96 |
| N: 27°19'14''  E: 55°21'40'' | 58 | 28 | Bandar Khmair | Animal living area (indoor) | Feb. 2017 | 2 | Female | 3 | [*Anopheles stephensi*](https://en.wikipedia.org/wiki/Anopheles_stephensi) | 97 |
| N: 27°19'14''  E: 55°21'40'' | 58 | 28 | Bandar Khmair | Animal living area (indoor) | Feb. 2017 | 1 | Female | 2 | *Anopheles dthali* | 98 |
| N: 27°19'14''  E: 55°21'40'' | 58 | 28 | Bandar Khmair | Animal living area (outdoor) | Feb. 2017 | 0 | Female | 2 | *Aedesvexans* | 99 |
| N: 27°19'14''  E: 55°21'40'' | 58 | 28 | Bandar Khmair | Animal living area (outdoor) | Feb. 2017 | 0 | Male | 1 | *Aedesvexans* | 100 |
| N: 27°19'14''  E: 55°21'40'' | 58 | 28 | Bandar Khmair | Animal living area (outdoor) | Feb. 2017 | 2 | Female | 2 | *Culisetalongiareolata* | 101 |
| N: 27°19'14''  E: 55°21'40'' | 58 | 28 | Bandar Khmair | Human living area (indoor) | Feb. 2017 | 0 | Female | 7 | [*Culexquinquefasciatus*](https://www.google.com/url?sa=t&rct=j&q=&esrc=s&source=web&cd=1&cad=rja&uact=8&ved=0ahUKEwj70rjt_r3ZAhUEKuwKHRuFCssQFggqMAA&url=https%3A%2F%2Fen.wikipedia.org%2Fwiki%2FCulex_quinquefasciatus&usg=AOvVaw39EYPbl6oFUCKPSf86_ABm) | 102 |
| N: 27°19'14''  E: 55°21'40'' | 58 | 28 | Bandar Khmair | Human living area (indoor) | Feb. 2017 | 0 | Male | 4 | [*Culexquinquefasciatus*](https://www.google.com/url?sa=t&rct=j&q=&esrc=s&source=web&cd=1&cad=rja&uact=8&ved=0ahUKEwj70rjt_r3ZAhUEKuwKHRuFCssQFggqMAA&url=https%3A%2F%2Fen.wikipedia.org%2Fwiki%2FCulex_quinquefasciatus&usg=AOvVaw39EYPbl6oFUCKPSf86_ABm) | 103 |
| N: 26°38'29''  E: 55°44'58'' | 37 | 22 | Bandar Khmair | Human living area (outdoor) | Feb. 2017 | 12 | Female | 40 | *Culexpipiens* complex | 104 |
| N: 26°38'29''  E: 55°44'58'' | 37 | 22 | Bandar Khmair | Human living area (outdoor) | Feb. 2017 | 0 | Male | 4 | *Culexpipiens*complex | 105 |
| N: 26°38'29''  E: 55°44'58'' | 37 | 22 | Bandar Khmair | Human living area (outdoor) | Feb. 2017 | 2 | Female | 10 | *Aedescaspius* | 106 |
| N: 26°38'29''  E: 55°44'58'' | 37 | 22 | Bandar Khmair | Human living area (outdoor) | Feb. 2017 | 0 | Male | 40 | *Aedescaspius* | 107 |
| N: 27°14'12''  E: 56°09'65'' | 39 | 24 | Bandar Abbas | Animal living area (indoor) | Feb. 2017 | 8 | Female | 20 | *Culexpipiens* complex | 108 |
| N: 27°14'12''  E: 56°09'65'' | 39 | 24 | Bandar Abbas | Animal living area (indoor) | Feb. 2017 | 0 | Male | 30 | *Culexpipiens* complex | 109 |
| N: 27°50'92''  E: 56°63'67'' | 34 | 23 | Bandar Abbas | Human living area (outdoor) | Mar. 2017 | 7 | Female | 50 | *Culexpipiens* complex | 110 |
| N: 27°50'92''  E: 56°63'67'' | 34 | 23 | Bandar Abbas | Human living area (outdoor) | Mar. 2017 | 0 | Male | 50 | *Culexpipiens* complex | 111 |
| N: 27°50'92''  E: 56°63'67'' | 34 | 23 | Bandar Abbas | Human living area (outdoor) | Mar. 2017 | 6 | Female | 25 | *Culisetalongiareolata* | 112 |
| N: 27°50'92''  E: 56°63'67'' | 34 | 23 | Bandar Abbas | Human living area (outdoor) | Mar. 2017 | 0 | Male | 20 | *Culisetalongiareolata* | 113 |
| N: 27°49'84''  E: 56°86'86'' | 31 | 18 | Bandar Abbas | Human living area (outdoor) | Mar. 2017 | 25 | Female | 65 | *Culexpipiens* complex | 114 |
| N: 27°33'16''  E: 56°26'51'' | 28 | 20 | Bandar Abbas | Human living area (outdoor) | Mar. 2017 | 1 | Female | 8 | [*Culextheileri*](https://www.google.com/url?sa=t&rct=j&q=&esrc=s&source=web&cd=1&cad=rja&uact=8&ved=0ahUKEwjhocz8_cfZAhWQDewKHTTQCIcQFggnMAA&url=http%3A%2F%2Fwww.mosquitocatalog.org%2Ftaxon_descr.aspx%3FID%3D17494&usg=AOvVaw05pYpqoY0FcPP2UEWddGMA) | 115 |
| N: 27°33'16''  E: 56°26'51'' | 28 | 20 | Bandar Abbas | Human living area (outdoor) | Mar. 2017 | 0 | Male | 20 | [*Culextheileri*](https://www.google.com/url?sa=t&rct=j&q=&esrc=s&source=web&cd=1&cad=rja&uact=8&ved=0ahUKEwjhocz8_cfZAhWQDewKHTTQCIcQFggnMAA&url=http%3A%2F%2Fwww.mosquitocatalog.org%2Ftaxon_descr.aspx%3FID%3D17494&usg=AOvVaw05pYpqoY0FcPP2UEWddGMA) | 116 |
| N: 27°33'16''  E: 56°26'51'' | 28 | 20 | Bandar Abbas | Human living area (outdoor) | Mar. 2017 | 0 | Female | 25 | *Uranotaeniaunguiculata* Edwards | 117 |
| N: 27°33'16''  E: 56°26'51'' | 28 | 20 | Bandar Abbas | Human living area (outdoor) | Mar. 2017 | 0 | Male | 15 | *Uranotaeniaunguiculata* Edwards | 118 |
| N: 27°38'24''  E: 55°53'46'' | 55 | 32 | Bandar Abbas | Human living area (outdoor) | Apr. 2017 | 12 | Female | 50 | *Culexpipiens* complex | 119 |
| N: 27°38'24''  E: 55°53'46'' | 55 | 32 | Bandar Abbas | Human living area (outdoor) | Apr. 2017 | 0 | Male | 60 | *Culexpipiens*complex | 120 |
| N: 27°38'24''  E: 55°53'46'' | 55 | 32 | Bandar Abbas | Human living area (outdoor) | Apr. 2017 | 4 | Female | 15 | [*Anopheles stephensi*](https://en.wikipedia.org/wiki/Anopheles_stephensi) | 121 |
| N: 27°38'24''  E: 55°53'46'' | 55 | 32 | Bandar Abbas | Human living area (outdoor) | Apr. 2017 | 0 | Male | 30 | [*Anopheles stephensi*](https://en.wikipedia.org/wiki/Anopheles_stephensi) | 122 |
| N: 27°49'84''  E: 56°86'86'' | 47 | 30 | Bandar Abbas | Animal living area (indoor) | Apr. 2017 | 23 | Female | 50 | [*Anopheles stephensi*](https://en.wikipedia.org/wiki/Anopheles_stephensi) | 123 |
| N: 27°49'84''  E: 56°86'86'' | 47 | 30 | Bandar Abbas | Animal living area (indoor) | Apr. 2017 | 0 | Male | 40 | [*Anopheles stephensi*](https://en.wikipedia.org/wiki/Anopheles_stephensi) | 124 |
| N: 27°49'84''  E: 56°86'86'' | 47 | 30 | Bandar Abbas | Animal living area (indoor) | Apr. 2017 | 5 | Female | 30 | [*Culexquinquefasciatus*](https://www.google.com/url?sa=t&rct=j&q=&esrc=s&source=web&cd=1&cad=rja&uact=8&ved=0ahUKEwj70rjt_r3ZAhUEKuwKHRuFCssQFggqMAA&url=https%3A%2F%2Fen.wikipedia.org%2Fwiki%2FCulex_quinquefasciatus&usg=AOvVaw39EYPbl6oFUCKPSf86_ABm) | 125 |
| N: 27°49'84''  E: 56°86'86'' | 47 | 30 | Bandar Abbas | Animal living area (indoor) | Apr. 2017 | 0 | Male | 40 | [*Culexquinquefasciatus*](https://www.google.com/url?sa=t&rct=j&q=&esrc=s&source=web&cd=1&cad=rja&uact=8&ved=0ahUKEwj70rjt_r3ZAhUEKuwKHRuFCssQFggqMAA&url=https%3A%2F%2Fen.wikipedia.org%2Fwiki%2FCulex_quinquefasciatus&usg=AOvVaw39EYPbl6oFUCKPSf86_ABm) | 126 |
| N: 27°76'20''  E: 56°34'15'' | 40 | 28 | Bandar Abbas | Human living area (outdoor) | Apr. 2017 | 15 | Female | 55 | *Aedescaspius* | 127 |
| N: 27°76'20''  E: 56°34'15'' | 40 | 28 | Bandar Abbas | Human living area (outdoor) | Apr. 2017 | 0 | Male | 42 | *Aedescaspius* | 128 |
| N: 27°76'20''  E: 56°34'15'' | 40 | 28 | Bandar Abbas | Human living area (outdoor) | Apr. 2017 | 20 | Female | 50 | *Anopheles fluviatilis* | 129 |
| N: 27°76'20''  E: 56°34'15'' | 40 | 28 | Bandar Abbas | Human living area (outdoor) | Apr. 2017 | 0 | Male | 50 | *Anopheles fluviatilis* | 130 |
| N: 27°12'56''  E: 55°87'84'' | 55 | 35 | Bandar Abbas | Animal living area (outdoor) | May 2017 | 10 | Female | 20 | *Culexpipiens* complex | 131 |
| N: 27°12'56''  E: 55°87'84'' | 55 | 35 | Bandar Abbas | Animal living area (outdoor) | May 2017 | 0 | Male | 20 | *Culexpipiens* complex | 132 |
| N: 27°12'56''  E: 55°87'84'' | 55 | 35 | Bandar Abbas | Animal living area (outdoor) | May 2017 | 17 | Female | 30 | *Aedescaspius* | 133 |
| N: 27°12'56''  E: 55°87'84'' | 55 | 35 | Bandar Abbas | Animal living area (outdoor) | May 2017 | 0 | Male | 30 | *Aedescaspius* | 134 |
| N: 27°12'56''  E: 55°87'84'' | 55 | 35 | Bandar Abbas | Human living area (outdoor) | May 2017 | 15 | Female | 40 | [*Culexquinquefasciatus*](https://www.google.com/url?sa=t&rct=j&q=&esrc=s&source=web&cd=1&cad=rja&uact=8&ved=0ahUKEwj70rjt_r3ZAhUEKuwKHRuFCssQFggqMAA&url=https%3A%2F%2Fen.wikipedia.org%2Fwiki%2FCulex_quinquefasciatus&usg=AOvVaw39EYPbl6oFUCKPSf86_ABm) | 135 |
| N: 27°12'56''  E: 55°87'84'' | 55 | 35 | Bandar Abbas | Human living area (outdoor) | May 2017 | 0 | Male | 40 | [*Culexquinquefasciatus*](https://www.google.com/url?sa=t&rct=j&q=&esrc=s&source=web&cd=1&cad=rja&uact=8&ved=0ahUKEwj70rjt_r3ZAhUEKuwKHRuFCssQFggqMAA&url=https%3A%2F%2Fen.wikipedia.org%2Fwiki%2FCulex_quinquefasciatus&usg=AOvVaw39EYPbl6oFUCKPSf86_ABm) | 136 |
